# Task-Specific Phosphonium Iongels by Fast UV-Photopolymerization for Solid-State Sodium Metal Batteries

**DOI:** 10.3390/gels8110725

**Published:** 2022-11-09

**Authors:** Luca Porcarelli, Jorge L. Olmedo-Martínez, Preston Sutton, Vera Bocharova, Asier Fdz De Anastro, Montserrat Galceran, Alexei P. Sokolov, Patrick C. Howlett, Maria Forsyth, David Mecerreyes

**Affiliations:** 1 POLYMAT, University of the Basque Country UPV/EHU, Joxe Mari Korta Center, Av. Tolosa 72, 20018 Donostia-San Sebastian, Spain; 2 ARC Centre of Excellence for Electromaterials Science and Institute for Frontier Materials, Deakin University, Melbourne 3216, Australia; 3 Oak Ridge National Laboratory, Chemical Sciences Division, Oak Ridge, TN 37831, USA; 4 Center for Cooperative Rersearch on Alternative Energies (CIC energiGUNE), Basque Research and Technology Alliance (BRTA), Parque Tecnológico de Alava, Albert Einstein 48, 01510 Vitoria-Gasteiz, Spain; 5 Department of Chemistry, University of Tennessee, Knoxville, TN 37996, USA; 6 Ikerbasque, Basque Foundation for Science, Maria Diaz de Haro 3, 48011 Bilbao, Spain

**Keywords:** iongel electrolyte, polymer electrolyte, sodium metal battery

## Abstract

Sodium metal batteries are an emerging technology that shows promise in terms of materials availability with respect to lithium batteries. Solid electrolytes are needed to tackle the safety issues related to sodium metal. In this work, a simple method to prepare a mechanically robust and efficient soft solid electrolyte for sodium batteries is demonstrated. A task-specific iongel electrolyte was prepared by combining in a simple process the excellent performance of sodium metal electrodes of an ionic liquid electrolyte and the mechanical properties of polymers. The iongel was synthesized by fast (<1 min) UV photopolymerization of poly(ethylene glycol) diacrylate (PEGDA) in the presence of a saturated 42%mol solution of sodium bis(fluorosulfonyl)imide (NaFSI) in trimethyl iso-butyl phosphonium bis(fluorosulfonyl)imide (P111i4FSI). The resulting soft solid electrolytes showed high ionic conductivity at room temperature (≥10^−3^ S cm^−1^) and tunable storage modulus (10^4^–10^7^ Pa). Iongel with the best ionic conductivity and good mechanical properties (Iongel10) showed excellent battery performance: Na/iongel/NaFePO_4_ full cells delivered a high specific capacity of 140 mAh g^−1^ at 0.1 C and 120 mAh g^−1^ at 1 C with good capacity retention after 30 cycles.

## 1. Introduction

Today, lithium ion batteries (LIBs) are the leading energy storage technology in the market of consumer electronics and electric mobility [1]. However, it is unlikely that LIBs alone can satisfy the demand for large-format energy storage due to the limited availability and the increasing price of lithium sources. Recent research is focusing on emerging post-lithium-ion batteries [2,3]. Multivalent ion batteries—such as magnesium, zinc, and aluminum—hold the theoretical advantage of transferring multiple charges by each ion, but the development of these technologies is still in an early stage [4]. On the other hand, sodium-ion batteries (SIBs) have gained increasing traction in academia and industry with few companies—such as Faradion (UK) and CATL (China)—near to market introduction. Sodium is a cheap and extremely abundant element that displays a very similar electrochemical behavior to lithium [5,6]. Nevertheless, SIBs still face some research challenges including lower energy densities than LIBs [4]. SIBs usually employ hard carbon anodes and carbonate-based electrolytes. Replacing hard carbon-negative electrodes with sodium metal ones could theoretically increase the energy density if suitable electrolytes for sodium metal are found. Super-concentrated ionic liquid (IL) electrolytes have been under extensive investigation due to their superior stability as electrolytes for sodium and lithium metal batteries [7,8]. While previous studies focused on pyrrolidinium ionic liquids, only recently has there been interest in phosphonium-based ionic liquids. For instance, Hilder et al. described an electrolyte based on 42%mol NaFSI in trimethyl iso-butyl phosphonium bis(fluorosulfonyl)imide (42% mol NaFSI in P1114iFSI) electrolytes for long lasting and stable sodium metal batteries [9,10]. Despite these advantages, IL electrolytes require a porous separator and are limited by the risks of leakage [11]. To overcome these issues and enable solid-state sodium batteries, the preparation of solid gel electrolytes (also known as iongel electrolytes) has become a very popular solution. This novel class of materials combines the unique electrolyte properties of ILs with the superior mechanical properties of polymers. Ionic conductivity is one of the most important parameters in determining whether a material is a good candidate for use as an electrolyte in a battery (ionic conductivity values of the order of 10^−4^–10^−3^ S cm^−1^ are normally required). On the other hand, the storage modulus is a measure of the mechanical properties of the material, which are reflected in the resistance of the electrolyte to dendrite growth. Along the same line, we recently demonstrated the excellent performance of iongels with sodium metal, which involves several polymer matrixes and a pyrrolidinium-type sodium ionic liquid electrolyte [12]. The goal of this work is to explore the fast UV photopolymerization method to prepare an iongel for an all-solid-state battery using a sodium-metal anode and triphylite NaFePO_4_ as the cathode material, which includes the high-performing phosphonium ionic liquid electrolyte.

In this work, fast UV photopolymerization of poly(ethylene glycol) diacrylate (PEGDA) in the presence of 42% mol NaFSI in P111i4FSI was used to prepare self-standing iongel electrolyte membranes for application in sodium metal batteries.

## 2. Results and Discussion

**Figure 1:**
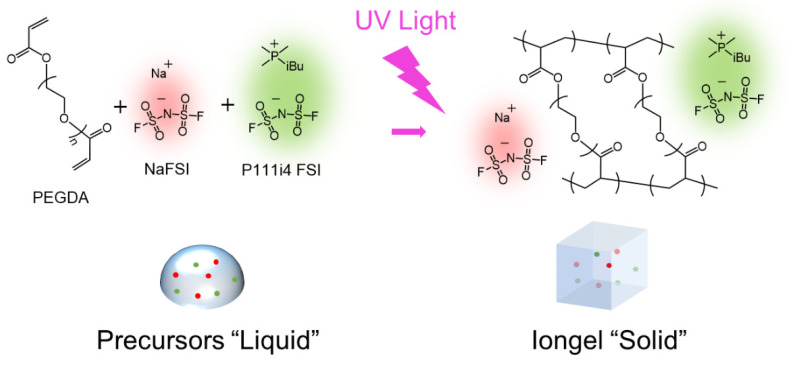
Synthetic representation of photopolymerization for the obtention of iongels.

Figure 1 shows the schematic diagram of the polymerization of cross-linked iongels. We used 2-hydroxy-2-methylpropiophenone (DAROCUR 1173) as a radical photoinitiator. We varied the amount of PEGDA cross-linker between 5 and 40 wt%, naming the iongel using the following codes: Iongel5, Iongel10, Iongel20, and Iongel40, where the number indicates the wt% of the PEGDA cross-linker. The obtained iongels were easy to handle, optically transparent, and did not leak the IL electrolyte. After polymerization, the soluble fraction was separated using a Soxhlet extractor and analyzed via ^1^H-NMR. The complete monomer conversion after the UV polymerization process was confirmed by the disappearance of the double bond signal associated with the acrylate function in the ^1^H-NMR; see Figure S1.

### 2.1. Dynamic Mechanical Analysis (DMA)

**Figure 2:**
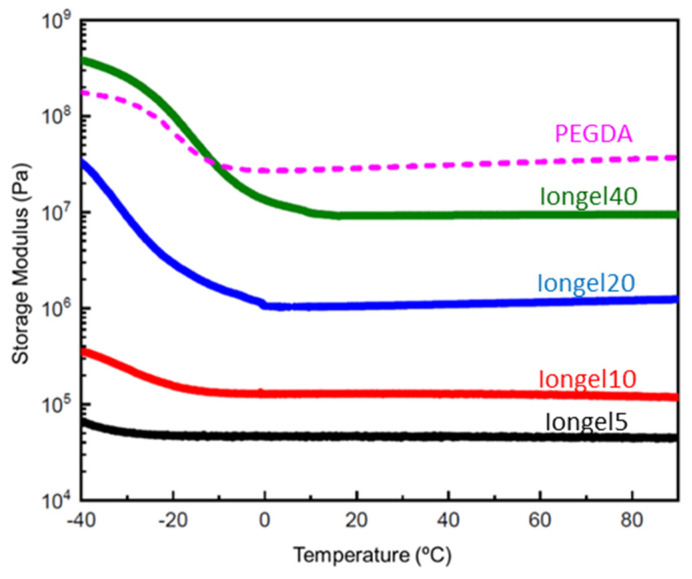
Storage modulus as a function of temperature for the different iongels.

DMA was used to determine the effect of PEGDA crosslinker content on the storage modulus of the phosphonium iongel electrolytes. Figure 2 shows the storage modulus as a function of temperature (measured between –40 and 90 °C), consisting of two characteristic regions. In the temperature region below 0 °C, a significant modulus change was assigned to the glass transition temperature (*T_g_*), and the *T_g_* increased with increasing PEGDA content. It should be noted that the transition region moved out of the measured temperature window at lower PEGDA content and no estimate of *T_g_* was possible from the DMA data. In polymers with small changes in heat capacity, it is more difficult to observe *T_g_* by DSC, while there is a large change in the storage modulus at *T_g_* [13].

Above room temperature, the rubbery plateau modulus increased with the PEGDA content in the electrolyte, having a maximum value of 9·10^6^ Pa with 40 wt% PEGDA, while the value of 100% PEGDA was 2.5·10^7^ Pa. This is a typical increase in the rubbery modulus with increasing crosslink density. Although it was possible to obtain a gel electrolyte with 5 wt% of PEGDA, these gels were very soft.

The storage modulus value at RT for Iongel40 was higher than those reported for other solvated ionic liquids (SIL) in polymeric hosts, e.g., PEO/80% SIL (viscous liquid) [14], PEGDA/79% SIL (420 kPa) [13], or PEGDMA/80% SIL (370 kPa) [15], and these results showed stable mechanical properties within the typical operational temperature range of solid-state batteries, i.e., RT and above.

### 2.2. Ionic Conductivity

**Figure 3:**
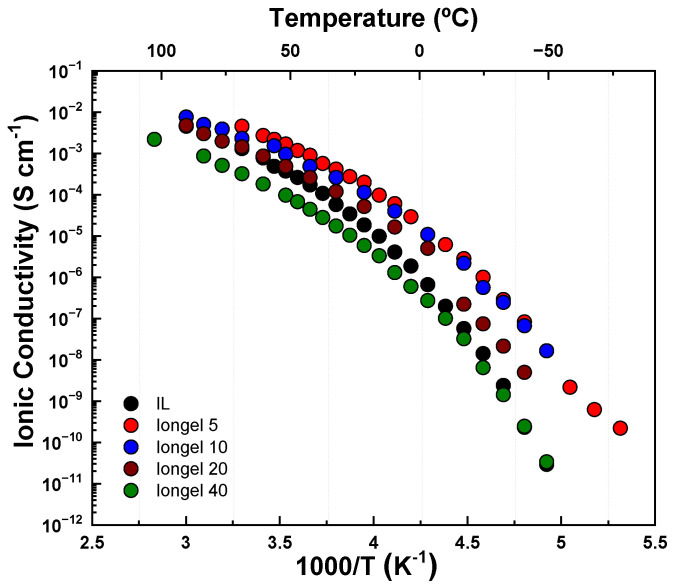
Ionic conductivity as a function of temperature for IL and iongels.

The ionic conductivity of the iongels was obtained from broadband dielectric spectroscopy (BDS) using the DC plateau from spectra in the conductivity representation. The IL was placed between two parallel plates made of brass and separated by a Teflon spacer ring with a thickness L = 100 μm. Figure 3 shows the plot of ionic conductivity between −80 and 80 °C. In general, a tradeoff was observed between ionic conductivity and the amount of PEGDA, and the sample with the lowest content of PEGDA (Iongel5) displayed the highest conductivity in the whole temperature range studied. The ionic conductivity of Iongel10 ranged between 7·10^−3^ S cm^−1^ at 80 °C and 2·10^−8^ S cm^−1^ at –70 °C. The conductivity decreased up to two orders of magnitude for higher PEGDA content, and the ionic conductivity of Iongel40 ranged between 2.5·10^−3^ S cm^−1^ at 80 °C and 3·10^−11^ S cm^−1^ at –80 °C. Interestingly, despite the significant differences of several orders of magnitude in the mechanical modulus, the conductivity only decreased by a factor of 3 in going from Iongel10 to Iongel40. Surprisingly, the pure ionic liquid electrolyte displayed a lower ionic conductivity than its cross-linked form at low polymer content, which may be due to the nanostructuration of the conductivity channels in the solid material.

### 2.3. Battery Cell Testing

**Figure 4:**
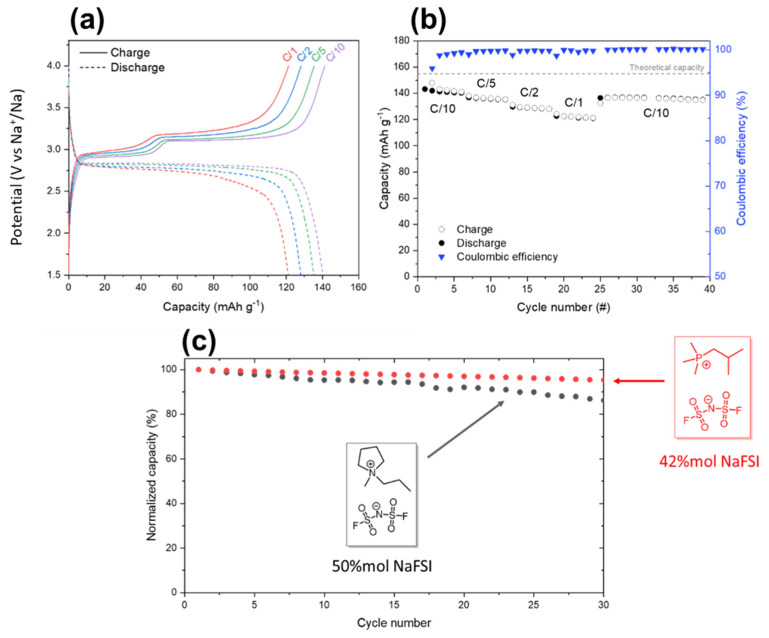
(**a**) Cycling behavior at 50 °C of a Na/iongel membrane/NaFePO_4_ cycled in the range of 1.5–4 V vs. Na^+^/Na at different C-rates; (**b**) specific capacity vs. cycle number plot; (**c**) normalized capacity as a function of cycle number for the electrolyte reported in this work (red points) and electrolyte previously reported by our group (black points) [12].

Finally, battery cells consisting of a sodium metal anode and a NaFePO_4_ cathode were assembled by sandwiching an Iongel10 membrane in between the electrodes. Figure 4a shows charge–discharge profiles at various C-rates extracted from a cell cycled in the range of 1.5–4 V vs. Na^+^/Na at 50 °C. As shown, the cell displayed two charge plateaus centered on 3 V vs. Na^+^/Na and a single sloping discharge profile. This asymmetric voltage is associated with the formation of an intermediate phase during the charge and is fully consistent with previously reported results of liquid and solid-state NaFePO_4_ cells [12,16,17,18,19]. At C/10, the cell delivered a maximum discharge capacity around 140 mAhg^−1^ corresponding to 90% of the theoretical capacity of NaFePO_4_ (154 mAh g^−1^). At higher current rates, the specific discharge capacities slightly decreased to 135 mAh g^−1^ at C/5, 130 mAh g^−1^ at C/2, and 120 mAh g^−1^ at C/1. Figure 4b shows the plot of specific capacities at various C-rates versus the cycle number and the corresponding coulombic efficiencies. During the first six formation cycles at low current rates (C/10), the coulombic efficiency increased rapidly to 99.5% and the cell delivered a stable discharge capacity. Even though the capacity at C/1 slightly decreased, the cell showed a good capacity retention and the coulombic efficiency remained close to 99.5%. Additionally, the cell recovered its initial capacity of ≈140 mAh g^−1^ with a coulombic efficiency of around 100%. In our previous work, a cell based on a N-propyl-N-methylpyrrolidinium bis(fluorosulfonyl)imide iongel delivered a maximum discharge capacity around 145 mAhg^−1^ at C/10, corresponding to 95% of the theoretical capacity of NaFePO_4_ (154 mAh g^−1^). Despite a slightly lower initial capacity, the phosphonium cell showed a far greater capacity retention, as shown by Figure 4c, of the normalized capacity of two cells cycling at C/10. In our previous work, we observed that iongels from superconcentrated phosphonium electrolytes exhibit better battery performance compared to the previously reported pyrrolidinium counterparts due to their superior electrochemical stability [12]. The results of this work suggest that the same behavior is observed in the iongel form of these electrolytes.

## 3. Conclusions

Four different iongel electrolytes were prepared by UV photopolymerization using trimethyl iso-butyl phosphonium bis(fluorosulfonyl)imide (P111i4FSI) ionic liquid and sodium bis(fluorosulfonyl)imide (NaFSI) salt, varying the amount of PEGDA in each from 5 to 40 wt%. The electrolytes showed an increase in storage modulus as the amount of PEGDA in the system increased, ranging from 10^4^ to 10^7^ Pa at 50 °C, for the electrolytes with 5 and 40 wt%, respectively. The ionic conductivity was measured using broadband dielectric spectroscopy (BDS) and obtained up to 10^−3^ S cm^−1^ at 50 °C; these values depended on the amount of polymer in each electrolyte. Taking into account these parameters, Iongel10 was selected for testing in a sodium battery, and this electrolyte showed a higher capacity retention compared to other pyrrolidinium-based electrolytes.

## 4. Materials and Methods

### 4.1. Materials

Trimethyl iso-butyl phosphonium bis(fluorosulfonyl)imide (P111i4FSI, Boron Molecular, Victoria, Australia) and sodium bis(fluorosulfonyl)imide (NaFSI, Solvionic, Toulouse, France) were dried under vacuum at 50 °C and transferred inside an Ar-filled glove box before use. Poly(ethylene glycol) diacrylate M_n_ 575 (PEGDA; Sigma-Aldrich, Madrid, Spain) was passed through a basic alumina column to remove the hydroquinone monomethyl ether inhibitor (MEHQ), filtered with a 0.45 μm syringe filter, and kept refrigerated at 5 °C before use. 2-hydroxy-2-methylpropiophenone (DAROCUR 1173, Sigma-Aldrich) was used as received.

### 4.2. Sample Preparation

A saturated 42% mol electrolyte solution of NaFSI in P111i4FSI was prepared inside an Ar-filled glovebox by stirring the solution on a hot plate at 50 °C, and stored inside the glovebox until use. A 2.5% wt monomer solution of DAROCUR 1173 in PEGDA was prepared outside the glovebox before use. Iongels membranes were prepared by mixing different weight amounts of the electrolyte and monomer solution (Table S1). The mixtures were cast on a silicone mold irradiated with a UV lamp for 90 s twice. The iongel membranes were kept for 24 h under vacuum at 90 °C and stored in an argon-filled glovebox until use. The membranes were circular disks (diameter = 14 mm; average thickness = 250 μm).

### 4.3. Physical–Chemical Characterization

DMA experiments were performed on a PerkinElmer DMA 8000 in tension mode with a heating rate of 5 °C min^−1^, at a 1 Hz frequency and strain of 25 µm, and in a N_2_ atmosphere. Broadband dielectric spectra in the frequency range of 10^−1^ to 10^6^ Hz were measured using a Novocontrol Concept-80 system, which includes an Alpha-A impedance analyzer and a Quatro Cryosystem temperature control unit. The samples were placed between the stainless-steel parallel plates with a 20 mm diameter, and the separation between the electrodes was determined by the film thickness, approximately 0.2 mm. The samples were placed inside the cryostat in a dry nitrogen atmosphere. The samples were equilibrated for at least 15 min after each temperature step to achieve thermal stabilization within 0.2 K.

The *Triphylite*-NaFePO_4_ cathode active material was synthesized using a two-step reaction reported previously [20].

### 4.4. Cell Assembly and Testing

Na/iongel/NaFePO_4_ cells were assembled for testing in sodium batteries. Sodium metal was used as the anode. The electrolytes and the electrodes were placed between two stainless-steel spacers (o.d. = 16 mm; thickness = 0.5 mm), and the cells were prepared inside the glovebox in argon atmosphere. These cells were measured in a VMP3 Biologic potentiostat at 50 °C and cycled at C/20 cycled in a potential range of 1.5–4 V for 30 cycles.

## Data Availability

Not applicable.

## Supplementary Materials

The following supporting information can be downloaded at: https://www.mdpi.com/article/10.3390/gels8110725/s1, Figure S1: H^1^-NMR spectra of soluble fraction after Soxhlet extraction; Table S1: Composition of the different electrolytes prepared.
