# Supplementary material for: Task-Specific Phosphonium Iongels by Fast UV-Photopolymerization for Solid-State Sodium Metal Batteries
Source: Gels. 2022 Nov 9;8(11):725. doi: 10.3390/gels8110725 (PMC9689987; doi:10.3390/gels8110725)
Supplement: Supplementary file 1 [file gels-08-00725-s001.zip › gels-2008110-supplementary.pdf]

Supplementary Information

Task-specific phosphonium iongels by fast UV photopolymerization for emerging solid state sodium metal batteries

Luca Porcarelli[1,2], Jorge L. Olmedo-Martínez[1], Preston Sutton[2], Vera Bocharova[5], Asier Fdz De Anastro[4], Montse Galceran[4], Alexei Sokolov[5], Maria Forsyth[1,2,6] and David Mecerreyes[1,6]*

[1]POLYMAT and University of the Basque Country UPV/EHU, Joxe Mari Korta Center, Av. Tolosa 72, 20018, Donostia-San Sebastian, Spain.

[2]ARC Centre of Excellence for Electromaterials Science and Institute for Frontier Materials, Deakin University, Melbourne, Australia

[3]Chemical Sciences Division, Oak Ridge National Laboratory, Oak Ridge, Tennessee 37831, United States.

[4] Center for Cooperative Rersearch on Alternative Energies (CIC energiGUNE), Basque Research and Technology Alliance (BRTA), Parque Tecnológico de Alava, Albert Einstein 48, 01510 Vitoria-Gasteiz, Spain.[5]Department of Chemistry, University of Tennessee, Knoxville, Tennessee 37996, United States

[6]Ikerbasque, Basque Foundation for Science, Maria Diaz de Haro 3, E-48011, Bilbao, Spain

*Corresponding author: david.mecerreyes@ehu.es

Table S1. Composition of the Different Electrolytes Prepared

	P111i4FSI	NaFSI	PEGDA	Darocur 1173
**Iongel5**	3.23 g	1.517 g	0.25 g	0.006 g
	64.6 wt%	30.3 wt%	5 wt%	0.1 wt%
**Iongel10**	3.062 g	1.438 g	0.5 g	0.013 g
	61.1 wt%	28.7 wt%	10 wt%	0.2 wt%
**Iongel20**	2.722 g	1.278 g	1 g	0.025
	54.2 wt%	25.4 wt%	19.9 wt%	0.5 wt%
**Iongel40**	2.042 g	0.958 g	2 g	0.05
	40.4 wt%	19 wt%	39.6 wt%	1 wt%

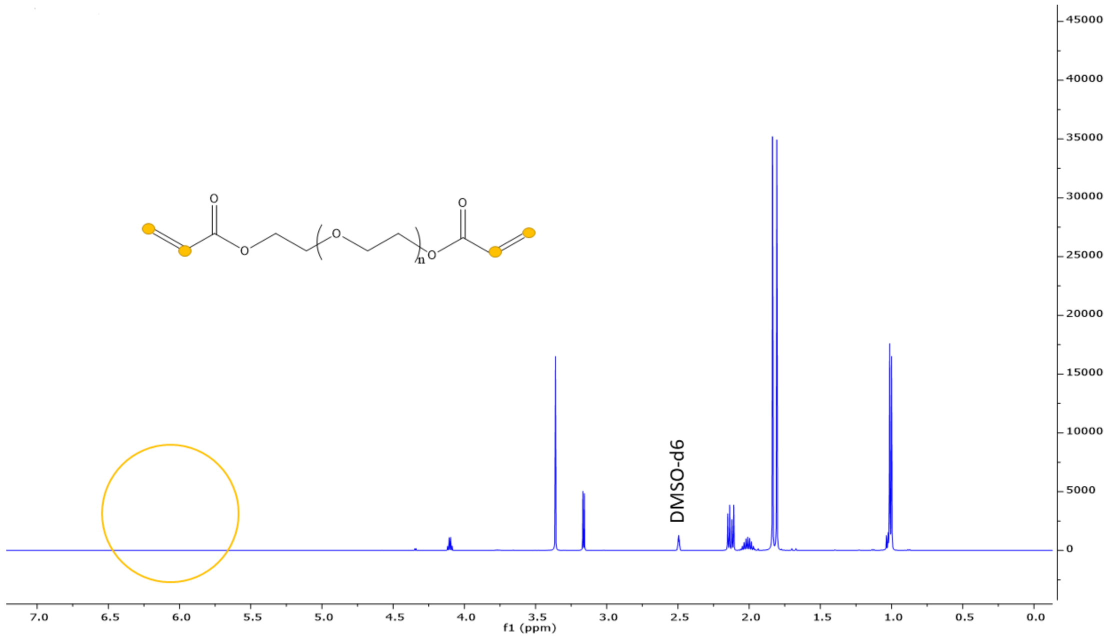

Figure S1. H^1-NMR spectra of soluble fraction after Soxhlet extraction.

Preparation of $NaFe_{0.8}Mn_{0.2}PO_4$

$NaFe_{0.8}Mn_{0.2}PO_4$ compounds were prepared from chemical sodiation of $Fe_{0.8}Mn_{0.2}PO_4$ using NaI as a reducing agent, according to the following reaction:

$$Fe_{0.8}Mn_{0.2}PO_4 + NaI \rightarrow NaFe_{0.8}Mn_{0.2}PO_4 + 1/2 I_2$$

For this, the $Fe_{0.8}Mn_{0.2}PO_4$ materials were stirred in acetonitrile with NaI taken in a 1:3 ratio under inert atmosphere and at 85ºC for two days. The suspension was then filtered, washed and dried in a vacuum oven overnight at 120ºC.

Preparation of $Na_{0.73}Fe_{0.2}Mn_{0.8}PO_4$ samples

$Na_{0.73}Fe_{0.8}Mn_{0.2}PO_4$ were obtained by mixing stoichiometric amounts of $NaFe_{0.8}Mn_{0.2}PO_4$ and $Fe_{0.8}Mn_{0.2}PO_4$ followed by heat treatment at 400ºC in Argon atmosphere.
